# Nurses’ Role in Nuclear Medicine Services: A Scoping Review Protocol (Part 1 of a Registered Report)

**DOI:** 10.3390/nursrep15110387

**Published:** 2025-10-31

**Authors:** Larissa Gleyciani Verdeli, Rosana Aparecida Pereira, Tatiana de Lourdes Gonzalez Sampedro, Leonardo Alexandre-Santos, Jennifer Machado de Oliveira, Michela Cristina Alves, Fernanda Raphael Escobar Gimenes, Lauro Wichert-Ana

**Affiliations:** 1 Nuclear Medicine and PET/C Laboratory, Department of Medical Imaging, Hematology and Clinical Oncology, Ribeirão Preto Medical School, University of São Paulo, Ribeirão Preto 14040-900, SP, Brazil; tlgonzalez@uce.edu.ec (T.d.L.G.S.); lalexandre@hcrp.usp.br (L.A.-S.); jennifermo@hcrp.usp.br (J.M.d.O.); michelaalves@hcrp.usp.br (M.C.A.); lwichert@fmrp.usp.br (L.W.-A.); 2 Department of General and Specialized Nursing, Ribeirão Preto College of Nursing, University of São Paulo, Ribeirão Preto 14040-900, SP, Brazil; rosana.pereira@usp.br (R.A.P.); fregimenes@eerp.usp.br (F.R.E.G.); 3 Facultad de Medicina, Universidad Central del Ecuador, Quito 170515, Ecuador

**Keywords:** nuclear medicine, radiology services, nursing, nursing team, multidisciplinary team, nursing care, radiopharmaceuticals, contrast media, radioactive tracers

## Abstract

**Background:** Nuclear medicine is a highly specialized field that combines advanced technology and multidisciplinary collaboration. Despite its complexity, the role of nurses in this context remains underexplored, especially regarding their clinical and administrative activities. **Methods:** This is a scoping review protocol developed according to the Joanna Briggs Institute (JBI) methodology and reported in accordance with the PRISMA-ScR guidelines, as recommended by the EQUATOR Network. The research question was structured using the PCC mnemonic (Population, Concept, and Context): What are the clinical and administrative activities performed by nurses in nuclear medicine services? A comprehensive search will be conducted in Web of Science, PubMed, Embase, Cochrane, SciELO, LILACS, Scopus, and CINAHL, complemented by grey literature sources such as Google Scholar and ProQuest Dissertations & Theses Global. No restrictions on language or publication date will be applied. Study selection and data extraction will be performed independently by two reviewers. This protocol is registered on the Open Science Framework (OSF) and is publicly accessible. The selection process will be detailed in a PRISMA-ScR flow diagram. A descriptive table will summarize the characteristics of the included studies, including the authors, year, country, study type, objectives, population, main nursing activities, and key findings. **Expected Outcomes:** The anticipated results are expected to clarify nurses’ contributions to patient safety and service quality in nuclear medicine. **Conclusions:** This review may also support the development of an assessment tool for nursing activities, guide professional training, and inform policies to strengthen nursing practice in this specialized field.

## 1. Introduction

Nuclear medicine is a key specialty within radiology and diagnostic imaging, widely utilized for both diagnosis and treatment involving radiopharmaceuticals [1,2]. Technological advancements have rendered diagnostic and therapeutic procedures in this field increasingly complex, demanding a highly qualified, multidisciplinary team to ensure patient safety and quality of care.

In this context, the role of nursing has gained prominence, as nurses play a crucial part in radiopharmaceutical administration, coordinating patient care workflows, and implementing safety protocols [3,4]. However, the specific contributions of the nursing team in nuclear medicine are underexplored in the literature, with this topic more commonly addressed in studies focusing on general radiology services [5]. Most research on the role of nursing in nuclear medicine concentrates on international contexts, with limited studies published in Brazil [6,7,8].

Nursing practice in conventional nuclear medicine, PET/CT, and radioisotope treatments (theranostics) encompasses the entire process, from initial assessment to post-procedure monitoring, including radiopharmaceutical administration and the prevention of adverse events [9,10,11]. The administration of radiopharmaceuticals, for instance, requires specialized knowledge to prevent adverse events, such as extravasation, which can compromise patient safety [12]. Despite increasing relevance, significant challenges remain, particularly concerning occupational radiation exposure, adherence to radiation protection protocols, and management of radiological emergencies. It’s important to note that nurses’ activities can vary depending on the country in which they work. In our country, nurses have greater autonomy in administering radiopharmaceuticals, providing direct patient assistance and intervention, and monitoring and guiding patients before and after exams.

The Brazilian National Nuclear Energy Commission (CNEN) regulation defines safety guidelines for professionals in nuclear medicine. However, the scientific exploration of implementing these guidelines in nursing practice remains limited [13]. The recognition of the nursing specialty in radiology and imaging by the Federal Nursing Council (COFEN) in 2020 reinforces the importance of this field. It highlights the need for research to systematize the activities performed by these professionals [14].

The continuous production of knowledge and adaptation to technological advancements are crucial for the dissemination and recognition of the nursing specialty in nuclear medicine. In this dynamic scenario, redefining nurses’ roles is essential to ensure patient safety and quality, and to promote evidence-based practice that aligns with sector demands [12].

Patient safety, defined as a set of practices and processes aimed at minimizing risks and reducing preventable harm [15], is a central aspect of nuclear medicine, as emphasized by the International Atomic Energy Agency (IAEA). In radiology and diagnostic imaging services, nurses play a strategic role in identifying and reporting adverse events, contributing to the improvement of care and management processes [12]. A factor that highlights the need for strategies that prevent failures and promote patient safety.

This article represents Part 1 of a Registered Report and presents the protocol for a scoping review designed to map the clinical and administrative activities of nurses in nuclear medicine services.

## 2. Justification

An initial exploratory search was conducted in the PubMed, Embase, CINAHL, Cochrane, Web of Science, SciELO/BVS, and LILACS databases using broad terms related to nuclear medicine and nursing. This search, limited to the past decade, retrieved a total of 773 documents: 60 from PubMed, two from SciELO, 483 from Embase, 16 from CINAHL, zero from the Cochrane Library, 165 from Web of Science, and 47 from LILACS/BVS. Analysis of the titles and abstracts revealed that few studies specifically address nurses’ activities in nuclear medicine, highlighting a gap in the literature that supports the relevance of this scoping review, as illustrated in Table 1.

Given the scarcity of studies systematically addressing the role of nursing in nuclear medicine, this scoping review aims to map the patient care and administrative activities performed by nurses in nuclear medicine services, identify knowledge gaps, and support the development of clinical practice guidelines [16].

The proposed scoping review aims to map the activities nurses perform in nuclear medicine services across both patient care and administrative contexts. This necessity emerged from the perceived gap in the scientific literature regarding the systematization of nursing’s role in this specific area.

To confirm this gap and justify the relevance of the present review, an exploratory search was conducted on the PROSPERO, Joanna Briggs Institute (JBI) [17], Open Science Framework (OSF) [18], and Web of Science databases. The objective was to identify previously registered scoping reviews, systematic reviews, or protocols addressing the topic. Searches were performed on 17 June 2025, using the following terms and descriptors: nursing, nurses, nuclear medicine, radiology department, combined with the terms “scoping review” and “systematic review,” as shown in Table 2.

No protocols or ongoing reviews were found on the PROSPERO, JBI, and OSF platforms. The 11 publications identified in Web of Science, after analysis of titles and abstracts, did not specifically address nursing activities within the nuclear medicine context.

The absence of prior reviews on this topic underscores the originality and necessity of this comprehensive, systematic mapping of available knowledge. This mapping can inform clinical practices and future guidelines for nursing performance in nuclear medicine services.

The expected outcome is the development of a specific tool to evaluate nurses’ patient care and administrative activities in nuclear medicine and PET/CT departments. This tool aims to validate its applicability in the nuclear medicine laboratory to support the hospital’s continuous quality program, assess the size of the nursing team based on their activities, and guide work schedules to avoid radiation overload for each team member, ensuring radiological and work safety. The findings of this review may also inform training programs and public policies aimed at enhancing and regulating nursing practice in this field. Thus, the scoping review is justified by the need to fill knowledge gaps regarding nursing practices in nuclear medicine services, thereby promoting evidence-based scientific and care advancements that align with sector demands.

## 3. Study Aim

To map and synthesize knowledge regarding the patient care and administrative activities performed by nurses in nuclear medicine services.

## 4. Methods

### 4.1. Note on Registered Report Format

This manuscript represents Part 1 of a Registered Report. It outlines the study protocol that will be implemented and subsequently submitted as Part 2 upon completion of the scoping review. The design, methods, and analyses described herein were established a priori to ensure transparency and reproducibility.

### 4.2. Study Design

This will be a scoping review, an approach indicated for underexplored areas or those with high study heterogeneity. It enables the identification of knowledge gaps, clarifies key concepts, and guides future investigations. The review will be conducted in accordance with the JBI Collaboration methodological guidelines, ensuring rigor and transparency. To ensure the quality and clarity in presenting results, the Preferred Reporting Items for Systematic Reviews and Meta-Analysis Protocols—extension for scoping reviews (PRISMA-ScR) checklist [19] will be used, as recommended by the EQUATOR Network. The protocol was registered on the Open Science Framework (OSF) platform [18]. It is available at the following link (https://osf.io/z8at6/) (accessed on 24 July 2025) and has generated the following DOI, DOI 10.17605/OSF.IO/Z8AT6, and any methodological adjustments will be documented in the final report.

The research question was defined using the PCC mnemonic (Population, Concept, and Context):

- P (Population): Nurses;
- C (Concept): Patient care and administrative activities performed by nurses in this context;
- C (Context): Nuclear medicine services.

Based on these elements, the research question is: “What are the patient care and administrative activities performed by nurses in nuclear medicine services?”

### 4.3. Definition of Concepts

#### 4.3.1. Patient Care Nursing Activities

These are direct actions focused on the patient to promote, maintain, or restore health. In the context of nuclear medicine, these steps include initial patient reception and clinical assessment, physical and emotional preparation for procedures, administration of radiopharmaceuticals or contrast media, monitoring for adverse reactions, providing post-examination care instructions, and ensuring safety throughout the process. Such activities require both technical and relational competencies, grounded in protocols and good clinical practices [12,14,20].

#### 4.3.2. Administrative Nursing Activities

These encompass actions of planning, organizing, coordinating, supervising, and evaluating the nursing team’s work processes. In nuclear medicine services, they may involve managing staff schedules, controlling and recording materials and radiopharmaceuticals, ensuring compliance with biosafety and radiation protection standards, interfacing with the multidisciplinary team, completing legal and technical forms, charting in patient records, and contributing to audits and accreditation processes. These activities underpin the quality, safety, and effectiveness of the services [13,14,20].

### 4.4. Eligibility Criteria

#### Types of Studies

This review will include primary studies, reviews, and guidelines that address nursing care and administrative activities performed by nurses in nuclear medicine services.

### 4.5. Exclusion Criteria

The following will be excluded from this scoping review:

- Studies that do not address the role of nurses or the nursing team in nuclear medicine.
- Publications that deal exclusively with technical, physical, or pharmacological aspects of nuclear medicine, without mention of nursing involvement.
- Studies that mention clinical and administrative activities carried out by nurses, but in other healthcare settings.
- Documents addressing general radiology or diagnostic imaging procedures not specific to nuclear medicine will be excluded.
- Works without full-text access, such as unpublished conference abstracts or incomplete records.
- Duplicate documents (when more than one version of the same study is identified, the most complete or most recent one will be retained).
- Studies that do not answer the research question.

### 4.6. Search Strategy and Data Extraction

The search strategy was developed in collaboration with a librarian from the University of São Paulo (Ribeirão Preto, Brazil). Descritores em Ciências da saúde da Biblioteca Virtual em Saúde (DeCS/BVS) and Medical Subject Headings (MeSH) will be used, covering databases such as Web of Science via Clarivate Analytics, PubMed via US National Library of Medicine, Embase via Elsevier, Cochrane, Latin American and Caribbean Health Sciences Literature (LILACS) via Biblioteca Virtual em Saúde (BVS), and Cumulative Index to Nursing and Allied Health Literature (CINAHL) via EBSCO.

Additionally, grey literature sources, including Google Scholar and ProQuest Dissertations & Theses Global, will be consulted. No language or time restrictions will be applied, and translations will be performed if necessary. References will be imported into EndNote, where duplicate records will be automatically removed, and subsequently, into Rayyan for title and abstract screening. Table 3 presents the search strategy developed for Medline via PubMed and then adapted for the other databases.

Two independent reviewers will examine the titles and abstracts of the retrieved articles from the search strategy to assess their eligibility for this study. In cases of disagreement, a third reviewer will be consulted to reach a consensus.

### 4.7. Data and Variables

The data to be extracted will include:

1. Publication Information: Authors, article title, year of publication, country of origin of the study, and journal/publication source.
2. Study Characteristics: Study type/methodology, objectives, sample size (when applicable), studied population (specifying the area of practice for health professionals with a focus on nuclear medicine), professionals’ experience level, and training received.
3. Concept—Nursing Activities: Focus on patient care and administrative activities, protocols used, and specific responsibilities in nuclear medicine.
4. Recommendations: Suggestions for future investigations and guidance for clinical practice in nuclear medicine.
5. Limitations: The authors acknowledge them, focusing on those pertinent to the nuclear medicine context.
6. Main Conclusions: Summary of the study’s most relevant conclusions, emphasizing essential aspects for the development of nursing practices in nuclear medicine.
7. Implications for practice: Summarize the impact of the findings for nurses’ professional practice in nuclear medicine.

### 4.8. Presentation of Results and Theoretical Framework for Data Extraction

The presentation of results will be structured according to the JBI Collaboration methodological recommendations, utilizing summary tables, traditional tables, and figures to facilitate the visualization and understanding of the extracted data.

- Summary tables will present the main characteristics of the included studies, such as author, year, country, study type, objectives, setting, main results, and implications for practice.
- A flow diagram following the PRISMA-ScR model will visually illustrate the study selection process.
- Figures and visual diagrams may also be used, when necessary, to represent the distribution of nursing activity categories across different nuclear medicine services or contexts.

Data extraction and categorization will be guided by the theoretical framework of Cruz and Gaidzinski (2013) [20], who proposed the development of an instrument for measuring nursing work time in a diagnostic imaging center. The model organizes nursing activities into three broad categories:

1. Direct Patient Care Nursing Activities: These include patient reception, preparation, monitoring, and guidance for diagnostic examinations and therapies.
2. Indirect Nursing Activities: These encompass charting in patient records, organizing materials, coordinating processes, and interfacing with the multidisciplinary team.
3. Personal or Support Activities: These are activities not directly linked to patient care but are part of the daily work routine (e.g., scheduled breaks, travel/movement).

This categorization will enable the clear identification of the type and nature of activities performed by nurses in nuclear medicine services, supporting the thematic mapping of evidence and the analysis of gaps in scientific knowledge.

## 5. Stage 2 Plans

Following the completion of the scoping review, Part 2 of this Registered Report will present the complete findings, including the descriptive synthesis of the included studies, thematic mapping, and discussion in light of existing evidence. The final manuscript will also address the methodological limitations and implications for nursing practice and policy development in nuclear medicine.

## 6. Expected Results and Anticipated Impact

As this paper represents Stage 1 of a Registered Report, no data collection or analysis have yet been performed. The anticipated results and impacts described below are hypothetical and based on the study’s objectives and methodological framework.

The selection of evidence sources will be summarized in a PRISMA-ScR flow diagram, showing the number of records identified, duplicates removed, titles and abstracts screened, full texts assessed for eligibility, included studies, and reasons for exclusion.

The characteristics of the included studies will be presented in a descriptive table including authors, year, country, study type, objectives, target population or context, reported nursing activities in nuclear medicine services, and main findings. This synthesis will provide an overview of the methodological, geographical, and thematic diversity in the literature on nurses’ roles in this field.

Although no findings are yet available, the anticipated results are expected to advance understanding of nursing practice in nuclear medicine. By mapping both clinical and administrative activities, this review will identify gaps in current knowledge, highlight opportunities for future research, and demonstrate how nurses contribute to patient safety and service quality in highly complex technological environments.

In addition, one of the expected outcomes is to support the development of an assessment tool for nursing activities in nuclear medicine and PET/CT laboratories, with potential applicability in daily practice. This contribution may strengthen evidence-based care, guide training initiatives, and inform health policies aimed at enhancing patient safety and the quality of nuclear medicine services, consolidating the essential role of nurses in this specialized area. In clinical practice, the nursing activity assessment tool will be used routinely in the nuclear medicine laboratory. The results of its application will be used in the hospital’s continuous quality program, allowing the assessment of the nursing team’s size based on their activities and guiding the organization of work schedules to avoid radiation overload for each team member. Finally, this tool will enable the identification and preservation of the quality of services provided to patients and of the nurse’s interventions.

## Figures and Tables

**Table 1:** Results of the initial exploratory search, 2025.

Database	Broad Search Strategy Used	Filters Applied	Results Found
PubMed	(“Nursing”[Mesh] OR “Nurses”[Mesh]) AND (“Nuclear Medicine”[Mesh] OR “Radiology”[Mesh])	Language: English, Portuguese; Period: 2015–2025	60
Scielo/BVS	(“medicina nuclear” OR “serviço de radiologia”) AND (enfermagem OR enfermeiros)	Language: Portuguese; Period: 2015–2025	2
Embase	(“nuclear medicine” OR “radiology department”) AND (nurses OR “nursing care”)	Language: English; Period: 2015–2025	483
CINAHL	(“nuclear medicine” OR “radiology”) AND (nurses OR “nursing care”)	Language: English; Period: 2015–2025	16
Cochrane Library	(“nuclear medicine” OR “radiology”) AND (nurses OR nursing)	Language: English; Period: 2015–2025	0
Web of Science	(“nuclear medicine” OR “radiology department”) AND (nursing OR nurses)	Language: English; Period: 2015–2025	165
LILACS	(“medicina nuclear” OR “radiologia”) AND (enfermagem OR enfermeiros)	Language: Portuguese; Period: 2015–2025	47

Note. Mesh = Medical Subject Headings; BVS = Biblioteca Virtual em Saúde. Search filters included language (English and Portuguese) and publication period (2015–2025).

**Table 2:** Results of the initial exploratory search specific to reviews, 2025.

Database/Platform	Search Date	Terms Used	Number of Results
PROSPERO	17 June 2025	nursing AND “nuclear medicine”	0
JBI	17 June 2025	nursing AND “nuclear medicine”	0
OSF	17 June 2025	nursing AND “nuclear medicine”	0

Note. PROSPERO = International Prospective Register of Systematic Reviews; JBI = Joanna Briggs Institute; OSF = Open Science Framework.

**Table 3:** Search strategy developed for the databases, 2025.

Database	Search Strategy
Medline via PubMed	(((“Nurses”[Mesh]) OR (“Nursing, Team”[Mesh]) OR (“Nursing Care”[Mesh]) OR (“Patient Care Team”[Mesh])) AND (((((“Health Services Administration”[Mesh]) OR (“Patient Care Management”[Mesh])) OR (“Nursing Process”[Mesh])) OR (“Models, Nursing”[Mesh])) OR (“Shared Governance, Nursing”[Mesh])) OR (“Nursing Services”[Mesh]) AND ((“Radiology Department, Hospital”[Mesh]) OR (“Nuclear Medicine”[Mesh]))
Scielo	(“Enfermeiras e Enfermeiros” OR Nurses OR “Equipe de Enfermagem” OR “Nursing, Team” OR “Cuidados de Enfermagem” OR “Nursing Care” OR “Equipe de Assistência ao Paciente” OR “Patient Care Team”) AND (“Administração de Serviços de Saúde” OR “Health Services Administration” OR “Gestão de Cuidados ao Paciente” OR “Patient Care Management” OR “Processo de Enfermagem” OR “Nursing Process” OR “Modelos, Enfermagem” OR “Models, Nursing” OR “Governança Compartilhada, Enfermagem “ OR “Shared Governance, Nursing” OR “Serviços de Enfermagem” OR “Nursing Services”) AND (“Serviço Hospitalar de Radiologia” OR “Radiology Department, Hospital” OR “Medicina Nuclear” OR “Nuclear Medicine”)
Embase	(‘nurses’/exp OR ‘nurses’ OR ‘nursing, team’/exp OR ‘nursing, team’ OR ‘nursing care’/exp OR ‘nursing care’ OR ‘patient care team’/exp OR ‘patient care team’) AND (‘health services administration’/exp OR ‘health services administration’ OR ‘patient care management’/exp OR ‘patient care management’ OR ‘nursing process’/exp OR ‘nursing process’ OR ‘models, nursing’/exp OR ‘models, nursing’ OR ‘shared governance, nursing’/exp OR ‘shared governance, nursing’ OR ‘nursing services’/exp OR ‘nursing services’) AND (‘radiology department, hospital’/exp OR ‘radiology department, hospital’ OR ‘nuclear medicine’/exp OR ‘nuclear medicine’)
CINAHL	(MH “Nurses+” OR MH “Team Nursing” OR MH “Nursing Care+” OR MH “Multidisciplinary Care Team+”) AND (MH “Health Services Administration+” OR MH “Managed Care Nursing” OR MH “Nursing Process+” OR MH “Nursing Models, Theoretical+” OR MH “Shared Governance, Nursing” OR MH “Nursing Service”) AND (MH “Radiology Service” OR MH “Nuclear Medicine”)
Cochrane	(MeSH “Nursing” OR MeSH “Nursing, Team” OR MeSH “Nursing Care” OR MeSH “Patient Care Team”) AND (MeSH “Health Services Administration” OR MeSH “Patient Care Management” OR MeSH “Nursing Process” OR MeSH “Models, Nursing” OR MeSH “Shared Governance, Nursing” OR MeSH “Nursing Services”) AND (MeSH “Radiology Service” OR MeSH “Nuclear Medicine”)
Web of Science	(“Nurses” OR “Nursing, Team” OR “Nursing Care” OR “Patient Care Team”) AND (“Health Services Administration”OR “Patient Care Management” OR “Nursing Process” OR “Models, Nursing” OR “Shared Governance, Nursing” OR “Nursing Services”) AND (“Radiology Department, Hospital” OR “Nuclear Medicine”)
LILACS/BVS	(“Enfermeiras e Enfermeiros” OR Nurses OR “Equipe de Enfermagem” OR “Nursing, Team” OR “Cuidados de Enfermagem” OR “Nursing Care” OR “Equipe de Assistência ao Paciente” OR “Patient Care Team”) AND (“Administração de Serviços de Saúde” OR “Health Services Administration” OR “Gestão de Cuidados ao Paciente” OR “Patient Care Management” OR “Processo de Enfermagem” OR “Nursing Process” OR “Modelos, Enfermagem” OR “Models, Nursing” OR “Governança Compartilhada, Enfermagem” OR “Shared Governance, Nursing” OR “Serviços de Enfermagem” OR “Nursing Services”) AND (“Serviço Hospitalar de Radiologia” OR “Radiology Department, Hospital” OR “Medicina Nuclear” OR “Nuclear Medicine”)
SCOPUS	(“Nurses” OR “Nursing, Team” OR “Nursing Care” OR “Patient Care Team”) AND (“Health Services Administration” OR “Patient Care Management” OR “Nursing Process” OR “Models, Nursing” OR “Shared Governance, Nursing” OR “Nursing Services”) AND (“Radiology Department, Hospital” OR “Nuclear Medicine”)
Google Scholar	(“Enfermeiras e Enfermeiros” OR Nurses OR “Equipe de Enfermagem” OR “Nursing, Team” OR “Cuidados de Enfermagem” OR “Nursing Care” OR “Equipe de Assistência ao Paciente” OR “Patient Care Team”) AND (“Administração de Serviços de Saúde” OR “Health Services Administration” OR “Gestão de Cuidados ao Paciente” OR “Patient Care Management” OR “Processo de Enfermagem” OR “Nursing Process” OR “Modelos, Enfermagem” OR “Models, Nursing” OR “Governança Compartilhada, Enfermagem” OR “Shared Governance, Nursing” OR “Serviços de Enfermagem” OR “Nursing Services”) AND (“Serviço Hospitalar de Radiologia” OR “Radiology Department, Hospital” OR “Medicina Nuclear” OR “Nuclear Medicine”)

Note. MeSH = Medical Subject Headings; MH = Major Heading (CINAHL thesaurus); BVS = Biblioteca Virtual em Saúde. Search strategies were adapted to each database’s controlled vocabulary and translated as needed.

## Data Availability

The study protocol is openly available in the Open Science Framework (OSF) at https://osf.io/z8at6/ (accessed on 21 August 2025).

## Public Involvement Statement

No public involvement in any aspect of this research.

## Guidelines and Standards Statement

This manuscript was drafted against the Joanna Briggs Institute (JBI) for scoping reviews methodology (17) and reported in accordance with the PRIS-MA-ScR guidelines (19).
